# Endodontic re-treatment and restorative treatment of a dens invaginatus type II through new technologies

**DOI:** 10.4317/jced.55840

**Published:** 2019-06-01

**Authors:** Álvaro Zubizarreta-Macho, Alberto Ferreiroa, Rubén Agustín-Panadero, Cristina Rico-Romano, Ana-Belén Lobo-Galindo, Jesús Mena-Álvarez

**Affiliations:** 1 DDS, PhD. Associate professor. Department of Endodontics. Faculty of Health Sciences. Alfonso X el Sabio University. Madrid. Spain; 2 DDS, PhD. Associate professor. Department of Dental Prosthesis. Faculty of Dentistry. Complutense University. Madrid. Spain; 3 DDS, PhD.Adjunct Professor. Department of Dental Medicine. Faculty of Medicine and Dentistry. University of Valencia. Spain; 4 DDS, MS. Associate professor. Department of Endodontics. Faculty of Health Sciences. Alfonso X el Sabio University. Madrid. Spain; 5 DDS, PhD. Head Director. Department of Endodontics. Faculty of Health Sciences. Alfonso X el Sabio University. Madrid. Spain

## Abstract

**Background:**

The complex anatomy of dens invaginatus makes access cavity to root canal system difficult, which has an impact on the prognosis of these teeth. A novel technique, based on new technologies, is proposed to make access cavity conservative and guided with minimal dental structure lost.

**Material and Methods:**

This case report shows the root canal retreatment and the endodontic surgery of a dens invaginatus type II in a left lateral upper incisor previously treated which was affected by a chronic apical abscess and an apical fracture. A Cone Beam Computed Tomography was performed to better diagnosis the dental anatomy. An intraoral scan was performed to get a digital 3D model. A computer-guided implant planning software was used to plan the access cavity and design the splint guided. Finally, the clinical crown was restored by a resin nanoceramic veneer made by a chairside system made up of an intraoral scanning unit and a grinding unit. Last, the authors carried through the endodontic surgery to extract the apical fractured fragment.

**Results:**

Follow-up appointments at 6, 12 and 18 months showed a radiographic reduction of the periapical lesion and absence of clinical signs.

**Conclusions:**

The splint guide allowed a guided and conservative access cavity to root canal system. It facilitates the root canal retreatment and improves the prognosis of the teeth with dental malformations.

** Key words:**CAD-CAM, Cone-Beam Computed Tomography, dens in dente, dens invaginatus, dental pulp cavity, endodontics.

## Introduction

*Dens invaginatus* (DI) is defined as a dental malformation with a variable prevalence range from 0.04% to 10% (1). Hülsmann M. describes its etiology as an invagination of the enamel organ into the dental papilla during the process of odontogenesis (DI *coronalis*) (2,3). Some authors focus the source of the etiology as an infolding of Hertwig’s epithelial root sheath into the developing root (DI *radicularis*). The result of this malformation is the most severe alteration and receives the following meanings: dens in dente, invaginated odontoma, dilated gestant odontoma, dilated composite odontoma, tooth inclusion and *dentoid in dente* (3-5).

This anatomic disorder is classified into three categories according to the degree of malformation, and its relation to the periodontal ligament (PDL): Type I: the invagination ends as a blind sac, confined to the crown (65,9%). Type II: the invagination extends apically beyond the external cementoenamel junction (CEJ), ending as a blind sac confined to the crown of the tooth (29,5%). Type III: the invagination extends beyond the CEJ ending in the lateral (III a) or apical (III b) foramen (4,6%). The pulp tissue presents in the DI, is rarely directly correlated to the main root of the canal (3,5).

The teeth most affected by this anatomical alteration are the maxillary lateral incisors (9%) that can present a bilateral occurrence of 43% (3,5-7).

Cone-beam computed tomography (CBCT) is considered an essential tool for the diagnosis, treatment planning and follow-up appointments of teeth affected by anatomical malformations (8-10). Moreover, the digital file obtained with CBCT can be combined with other digital files obtained from other devices, as for example extraoral scanner or digital, for to integrate the information for manufacturing splint guides for different treatments, even in the field of endodontics. In addition, to restore the tooth treated endodontically, it is possible to do chairside digital workflow, through of intraoral digital scanner and milling unit. Within of this chairside workflow, it is possible to do a the restoration with a total chairside workflow, where the clinician to design and to fabricate the restoration in the clinic or partial chairside workflow, where an expertise lab technician receive the standard triangulation language (STL) file, via internet and to design the restoration and to sent the STL file of desing to the clinic for manufacturing. Within of this chairside workflow, it is possible to do a the restoration with a total chairside workflow, where the clinician to design and to fabricate the restoration in the clinic or partial chairside workflow, where an expertise lab technician receive the standard triangulation language (STL) file, via internet and to design the restoration, and after to sent the STL file of the design of the restoration, to the clinic for manufacturing.

The complex and variable anatomy from DI influences over the treatment planning, making unpredictable conventional therapeutic procedures such as: preventive treatments (1,3,8), nonsurgical root canal therapy (4,11), endodontic surgery (12,13), pulp revascularization (14-16), intentional replantation, extraction (17), or a combination of these (1-3). The management of traumatized teeth with a chronic apical abscess associated to fractured apex requires a multidisciplinary approach with endodontic surgery as an adjunct of root canal therapy in order to promote bone healing (18).

Conventional location of root canal system of DI results difficult, and has the risk to cause perforations, fractures or weak the tooth. Furthermore, the complex and irregular anatomy of DI make any canal susceptible of being omitted, leading to endodontic treatment failure (19). Therefore, is important to consider an access cavity procedure able to locate all canals without removing excess tooth structure. The purpose of this case report was to show an innovative technique to allow a guided and conservative access cavity to the root canal system of a DI type II, preserving dental tissue as much as possible and making the therapeutic prognosis more predictable.

## Case Report

**Figure 1:**
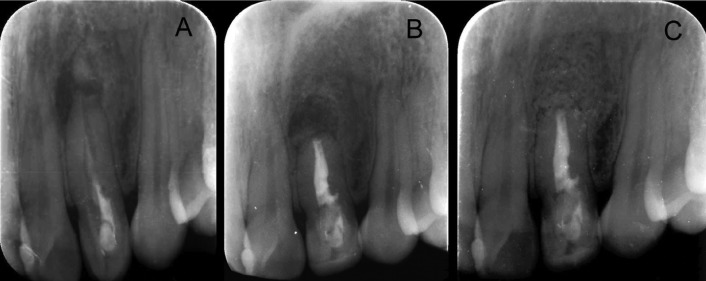
Preoperative periapical radiograph of tooth 2.2 showing previous endodontic treatment and the apical fracture (arrow) (A), post-surgery periapical radiography (B) and check periapical radiography follow-up at 18 months after treatment. Complete periradicular healing (C).

**Figure 2:**
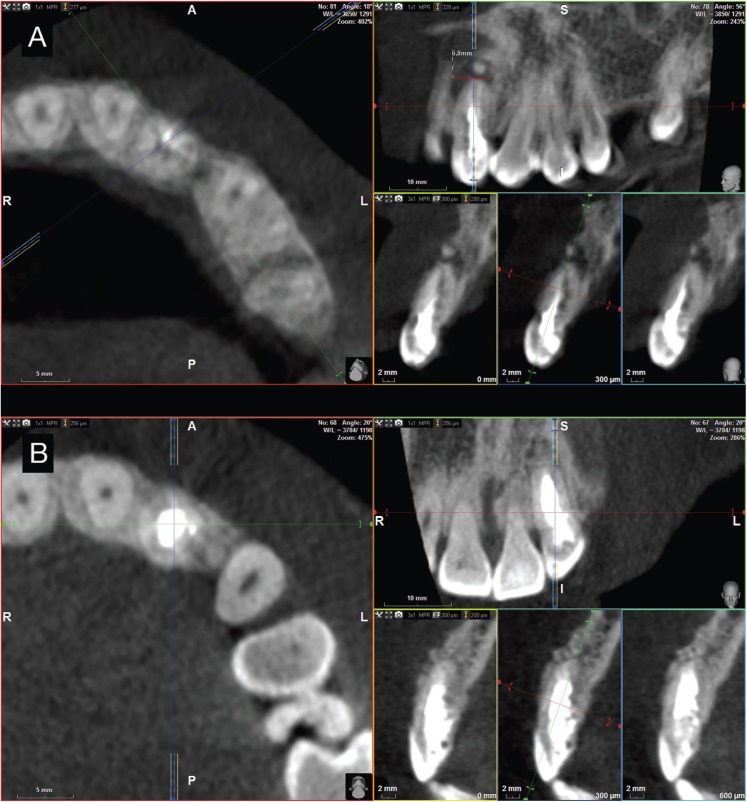
Preoperative cone-beam computed cross-sections. Notice apical fracture and gutta-percha presence (A). Cone-beam computed tomography cross-sections follow-up at 18 months after treatment. Complete periradicular healing (B).

**Figure 3:**
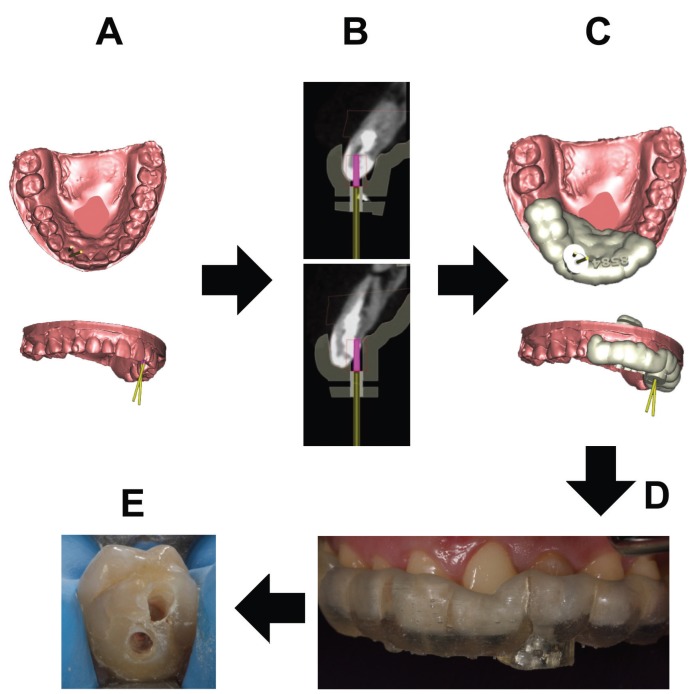
Digital workflow to two access cavity (A-E).

A 28-year-old female patient was referred to the department of Endodontics of the Alfonso X el Sabio University, for diagnosis and management of a chronic apical abscess on a previously treated tooth, secondary to a traumatism on left upper lateral incisor (20) (Fig. 1A). A CBCT (WhiteFox, Acteón Médico-Dental Ibérica S.A.U.-Satelec, Merignac, France) was taken to better diagnosis the complexity of the DI Oehlers type II and the fractured apex, based on the following exposure parameters: 105,0 kilovolt peak, 8,0 milliamperes, 7,20 seconds, and a field of view of 15x13 millimeters (Fig. 2A). The anatomical complexity of root canal system, justified the need to request a splint that would guide the access cavities. The design of the splints was assisted by means of a computer-guided implant planning software (SimPlant®, Dentsply Implants, Hasselt, Belgium). A 3D printer (ProJet® 6000. 3D Systems©, Rock Hill, SC, USA) was used to fabricate the splints from stereolithography resin, with the exception of the stainless steel cylinder used to guide the access cavities to root canal system (Fig. 3). The length and the diameter of the guiding cylinders were 5 mm y 1,3 mm, respectively. A diamond burr surface was selected with a diameter of 1,2 mm on the active part, and a total length 14 mm (Ref.: 882 314 012, Komet Medical, Lemgo, Germany).

Root canal retreatment was performed, after applying infiltrative anesthesia (Artinibsa, Inibsa S.A., Lliça de Vall, Barcelona, Spain) and rubber dam for absolute isolation (Hygenic® dental dam, Coltene® Whaldent Gruppe, Altstätten, Switzerland). The access cavities were made by means of the splint guide (Fig. 3), and the working length was established by an electronic apex locator (Root ZX, Morita, Tokyo, Japan). The root canal system was cleaned and shaped with Reciproc® endodontic rotary system (R25, VDW®, Munich, Germany). The root canal system was copiously irrigated with 5,25% sodium hypochlorite, and the contact between the irrigating solution and the surface of the root canal walls was enhanced by using an ultrasonic tip (IRRI S, VDW®, Munich, Germany) (21). After drying the root canal system with sterile paper points (Dentsply Maillefer, Ballaigues, Switzerland), the root canal system was sealed by employing a warm gutta-percha system (Calamus, Dentsply Maillefer, Ballaigues, Switzerland) and a epoxy resin-based cement (AH Plus, Dentsply DeTrey, Konstanz, Germany). The access cavities were filled with a composite restoration (Filtek Supreme XTE, 3M™, MN, USA).

**Figure 4:**
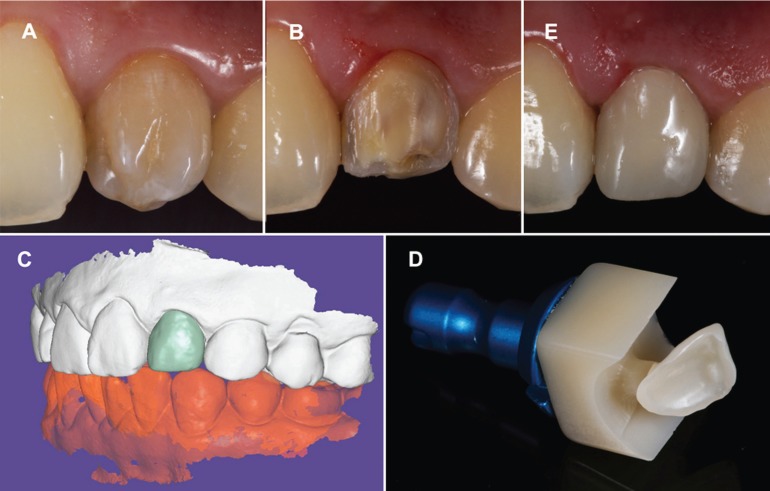
Treatment planning sequence of CAD/CAM veneer (A-E).

Then, the tooth was prepared for an indirect veneer, for improving the aesthetic. It was made with a digital worklow,from an impression made through an intraoral scanner (True Definition Scanner, 3M™, MN, USA). The Stl file obtained, with this device, was sent over the internet, to a expertise dental technician, for designing the restoration through a CAD software (ExoCad, Darmstadt, Germany). Once the restoration was designed, it was sent to the dental clinic for manufacturing the indirect veneer with a block of resin nano- ceramic material (Lava™ Ultimate A2 HT, 3M™, MN, USA) in a 4-axis milling unit (Roland®DG Iberia DWX-4, Hamamatsu, Japan). The restoration was cemented with a complete isolation with rubber dam , using a light-curing cement (RelyX™ Veneer™ TR, MN, USA) (Fig. 4).

The endodontic surgery was carried out in order to remove the fractured apex. A modified Newman incision was made to remove the cystic tissue through the curettage of the bone defect. A 3mm retrocavity was designed by means of a diamond ultrasonic tip (Ref.: PUSURG #2, ProUltra®, Dentsply Maillefer®, Ballaigues, Switzerland). An haemostatic agent of aluminum chloride-based was used (Expacen®, Acteon® Pierre Roland, Merignac, France) and the retrocavity preparation was sealed with mineral trioxide aggregate as root-end filling material (ProRoot® MTA white, Dentsply Tulsa Dental®, Tulsa, UK). Then, a bone graft was carried out (Bio-Oss®, Geistlich Pharma North America Inc., Princeton, NJ, USA) and the incision was sutured with nylon 5/0 (Figure 5) (Seralene®, Serag-Weissner Iberia, Madrid, Spain) (Fig. 1B). The patient was scheduled for follow-up appointments at 6, 12 and 18 months in order to assess the evaluation of the treatment. At the 18 months follow-up visit, the patient remained asymptomatic and periapical radiograph (Fig. 1C) and CBCT scan (Fig. 2B) showed a reduction of the periapical lesion. The size of the periapical lesion was measured over the CBCT using the ruler tool of the WhiteFox software. Preoperative lesion (Fig. 2A) showed a 6,8 mm diameter in its higher plane, which was disappeared at 18 months follow-up (Fig. 2^2^B).

## Discussion

Treatment planning of dental malformations is directly related to the anatomical complexity of each individual case. Many therapeutic strategies have been proposed to treat DI, depending on the previous diagnosis. Nevertheless, none focused attention on the access cavity complicating the restorative treatment procedure and weakening the coronal structure (19). The complex anatomy associated to this type of DI previously treated with fractured apex, required advanced therapeutic procedures which can be a challenge for the clinician. A straight line access to the root canal system is necessary to locate all canals, facilitate disinfection and complete debridement, as well as for avoiding loss of an excess of dental structure (20-22). One narrow single bur used to the access cavity implies a higher accuracy and a more conservative preparation than several burs of different shape. The guided access devices of computer-aided rapid prototyping were designed to apply them in implant guided surgery (23). However, their high versatility has pushed their application in endodontic field, enabling the location of the root canal systems affected by anatomical malformations (24) or calcific metamorphosis (25,26), through guided and conservative access cavities. This CAD-CAM access devices show a mean deviation of 0,16 mm between the planned and performed access cavity at the tip of the bur (22,23). These measures allowed an accurate, conservative and safe access cavity to the root canal system. The manufacturing process of the guided access devices may be carried out using two computer-aided rapid prototyping additive techniques: stereolithography or 3-dimensional digital printing. Nevertheless, the computer-aided rapid prototyping models obtained from 3-dimensional digital printing technology (ProJet® 6000. 3D Systems©, Rock Hill, SC, USA), show a degree of accuracy and fit higher to the models obtained from stereolithography technology (27), reducing the discrepancies noted at the access for implant guided surgery23 and allowing the location of the root canals (22).

It was considered that the fractured apex should be removed because it was not possible to clean the root canal present inside, and could promote the persistence of the infectious process (19). CBCT imaging was used as radiographic method for the diagnosis, treatment planning and follow-up appointments, because it has showed a more accurate in identifying root canal system than periapical radiograph (9). This radiographic tool gives 3-dimensional digital images enabling to analyze root canal system, including the bone affected area with significatively less radiation exposure tan computed tomographic scans. This radiographic tool gives 3-dimensional digital images enabling to analyze bone healing process with significatively less radiation exposure tan computed tomographic scans (8). Therefore, it was used as adjunct of clinical examination to assess the treatment outcome in the follow-up appointments.

For the final restorative treatment of the tooth a monolithic indirect veneer was obtained. A digital workflow was used in this case, by using an intraoral digital scanner and milling unit, avoiding to work with a physic model (28). We used in this case a chairside workflow, where the restoration is placed in one appointment. In our case, we use partial chairside system, where our restoration was designed by an expertise dental technician with a lab CAD software, with more options and applications than the CAD software of the devices of dental clinics, obtaining a more detailed design. Nevertheless, this workflow with sending the STL file to the dental technician, increase the treatment time with respect a total chairside system, where all steps are done in dental clinic.

These type of restorations obtained from a digital impression seem to have a similar o better results in several studies where the marginal of the restorations, were better to compare with a conventional workflow (29-31). Also in these type of cases, where a digital workflow with monolithic restorations, the result of clinical parameters such as points of contact, occlusion and the fit at clinical level are better than those restorations manufactured with a conventional workflow. In the clinical case presented, no adjustments had to be made in the areas of interproximal contact and the adjustment of the restoration was adequate, in accordance with the existing scientific literature.

## Conclusions

CBCT is the most effective diagnostic method to know the internal anatomy of the teeth with anatomical malformations, and perform the radiographic follow-up. The computer-guided implant planning software is an effective way of planning the root canal treatment. The splints made by 3-dimensional digital printing allow us a accurate, conservative and safe cavity access from the teeth affected by anatomical malformations. The chairside systems allow us to realize precise and foreseeable restorations in a single clinical session.
